# Analysis of Vibration-Damping Characteristics and Parameter Optimization of Cylindrical Cavity Double-Plate Phononic Crystal

**DOI:** 10.3390/ma16134605

**Published:** 2023-06-26

**Authors:** Chunsheng Song, Qi Yang, Xuechun Xiong, Rui Yin, Bo Jia, Yaru Liang, Haining Fang

**Affiliations:** 1 School of Mechanical and Electrical Engineering, Wuhan University of Technology, Wuhan 430070, China; song_chsh@163.com (C.S.);; 2 School of Management, Wuhan University of Technology, Wuhan 430070, China

**Keywords:** local resonance phononic crystal, double-layer plate structure, vibration transmission test, RSM, PSO

## Abstract

For the application of low-frequency vibration damping in industry, a cylindrical cavity double-layer plate-type local resonance phononic crystal structure is proposed to solve low-frequency vibration in mechanical equipment. Initially, using COMSOL 5.4 software, the bending wave band gap is calculated in conjunction with elastic dynamics theory and the BOLOCH theorem to be 127–384 Hz. Then the mechanism of bending wave gap is analyzed by combining element mode shape and an equivalent model. Subsequently, the bending vibration transmission characteristics of the crystal plate are explained, and the vibration-damping characteristics are illustrated in combination with the time–frequency domain. An experimental system is constructed to verify the vibration-damping properties of crystal plates; the experimental results and simulation results are verified with each other. Finally, the element structural parameters are optimized using the RSM. Fifty-four sets of experiments are designed based on six structural factors and three levels, and the expressions between the bending wave band gap and six structural factors are obtained. Combining the particle swarm algorithm, the optimization is performed with the band gap width as the target. This method is shown to be more accurate than the commonly used interior point method. The structure of cylindrical-cavity-type phononic crystal and the parameter optimization method proposed in this paper provide a certain reference for the design of local-resonance-type phononic crystal.

## 1. Introduction

Recently, science and technology have experienced rapid progress, and the machinery industry has also grown by leaps and bounds. However, mechanical vibration has remained a significant problem in the operation of mechanical equipment. Excessive vibration can disrupt equipment operation, causing noise hazards. Low-frequency mechanical vibration exists in many engineering applications, such as ships, buildings, and large equipment, and it is closely related to people’s lives. Mechanical vibration not only reduces the safety of equipment operation but also poses hidden risks to people’s property and life safety [1,2,3,4]. There are two common methods of vibration reduction—isolation and absorption—which work based on different modes of action. Phononic crystal structures are further expanded on the basis of traditional periodic structures and are new methods and concepts for achieving vibration and noise suppression [5,6]. The elastic wave band gap formed by the phononic crystal structure exhibits good suppression and attenuation effects on vibrations in the frequency band [7,8].

The band gaps of phononic crystals can be divided into Bragg-scattering-type and local-resonance-type [9,10]. The range of the Bragg-type band gap is closely related to the size of its geometric structure, making it impractical to obtain a low-frequency band gap suitable for engineering applications in small structural sizes. The characteristics of the local-resonance-type can compensate for this disadvantage. By reasonably designing structures, lower-frequency range band gaps can be obtained to cope with vibration and noise reduction problems in industry [11,12,13]. There are many structures for local-resonance-type phononic crystal, among which convex phononic crystals have been proven to generate low-frequency band gaps as a typical example [14,15,16,17,18,19,20,21,22]. Ravanbod M [23] designed a cross-shaped light scattering structure based on a square lattice metamaterial, and the results showed that it produced multiple broadband gaps. LI et al. [24] designed a single-sided periodic ring resonant radial localized resonant phononic crystal structure, which has better vibration reduction performance compared to traditional industrial boards. Liu H et al. [25] designed various helical phononic crystal structures and completed structural optimization. Due to their excellent vibration reduction performance, phononic crystal structures have been developed into vibration reduction and sound insulation components for using in engineering scenarios and equipment [26], including transportation [27,28], shipbuilding and ocean engineering [29,30], aerospace [31], and other fields in which phononic crystal structures can play a crucial role. In the field of emerging materials, phononic crystals and their related research were carried out, and an ultra-sensitive gas sensor was made using phononic crystals [32]. In addition, research on combining phononic crystals with carbon fiber materials has also yielded some achievements [33,34].

The band gap range of the phononic crystal structure is closely linked to its unitary parameters. To better understand this relationship and facilitate the optimization of structural parameters, scholars commonly employ response surface methodology (RSM) analysis to examine the connection between variables and band gaps, but optimization using the interior point method tends to be trapped in a local optimum solution [35,36,37]. Furthermore, some researchers have used alternative approaches to optimize phononic crystal structures. Zhang BQ et al. [38] trained phononic crystal structure models using neural networks, while Chen LY et al. [39] utilized genetic algorithms to optimize parameters.

Aiming at low-frequency vibration industrial applications, a cylindrical cavity double-layer plate phononic crystal structure is designed to address low-frequency vibration in mechanical devices. The proposed structure is analyzed for its vibration damping characteristics using a combination of finite element method and experiments. A structural parameter optimization scheme is established through RSM to obtain a relatively optimal parameter combination. Firstly, the design of the cylindrical cavity double-layer plate phononic crystal elements is presented, followed by an analysis of the band gap range and its formation mechanism. Its vibration damping performances are further verified using vibration transmission characteristic analysis. Secondly, an experimental platform is built to validate its vibration-damping performances. Finally, RSM analysis is carried out using 54 sets of orthogonal experiments to obtain the expressions between the bending wave band gap and six structural parameters. A nonlinear programming problem is constructed to obtain the maximum band gap width and corresponding structural parameters. The particle swarm algorithm is employed to deal with the nonlinear problem, resulting in the maximum band gap width and corresponding structural parameters.

## 2. Cylinder Cavity Double-Layer Plate Phononic Crystal

### 2.1. Method

In general, phononic crystal structures’ medium characteristics are described using elastic dynamics theory, and to further simplify the solution range, lattice energy theory and the BLOCH theorem are utilized. Below is a brief explanation of the theoretical methods and related formulas mentioned above.

In a homogeneous medium with linear elasticity and isotropy, by solving the three basic equations of force, displacement, and stress for a specific element in a uniform, the elastic dynamics equation can be obtained and converted into vector form:

(1) $$\rho \ddot{u}=\rho f+\left(\lambda +\mu \right)\nabla \left(\nabla \cdot u\right)+\mu \nabla^{2}u$$

In the equation, $u\left(x\right)$ is the particle displacement vector, ∇ is a Hamiltonian operator, ρ is the density, μ and λ are Lamé constants, and ω is the circular frequency.

Since phononic crystals have spatial periodicity, lattice and band theory is used to describe the periodicity of the structure, and the basis vector is defined as:

(2) $$R_{n}=n_{1}a_{1}+n_{2}a_{2}+n_{3}a_{3}$$

where $\left\{a_{1},a_{2},a_{3}\right\}$ is a set of linearly independent vectors; $n_{1},n_{2},n_{3}$ are positive integers; and any point in the phononic crystal structure can be represented using primitive cells and basis vectors. The spatial periodicity and symmetry of phononic crystals allow for the representation of the system’s eigenfields according to the BLOCH theorem as follows:

(3) $$u\left(x\right)=u_{k,n}\left(x\right)e^{ik\cdot x}$$

k is the wave vector, and the amplitude modulation function $u_{k,n}(x)$ also affects the lattice vector $R_{n}$ has translational periodicity, which satisfies:

(4) $$u_{k,n}\left(x+R_{n}\right)=u_{k,n}\left(x\right)$$

The BLOCH theorem indicates that the eigenfield of a phononic crystal can be expressed by a wave vector within the first Brillouin zone. Thus, by calculating the eigenfield for all wave vector values in this zone, the analysis of the system’s eigenfield can be completed, and the range of solutions can be reduced accordingly.

The finite element method is a commonly used technique for solving the energy band structure of phononic crystal structures. Initially, the whole model is discretized into small elements, and then the relationships between the displacement and stress of these elements are combined to obtain global equations and boundary constraints. Finally, numerical calculations and analyses are performed. The equilibrium equation of the structure is given as follows:

(5) $$\left(K-\omega^{2}M\right)U=F$$

In Equation (5), F is load matrix, K is stiffness matrix, and M is mass matrix.

Finally, it is only necessary to introduce periodic boundary conditions so that any point in the structure satisfies the BLOCK boundary condition, namely:

(6) $$u\left(r+a\right)=u\left(r\right)e^{i\left(k\cdot a\right)}$$

In Equation (6), a means the lattice constant of structure.

### 2.2. Structure Analysis

**Figure 1:**
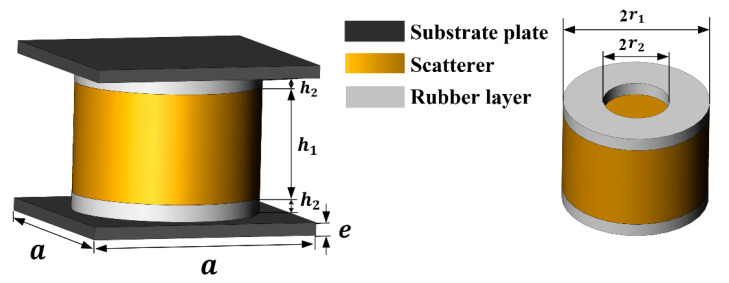
Phononic crystal unit structure.

For low-frequency mechanical vibration, local resonance phononic crystal structures are commonly designed to obtain a large band gap width and lower initial frequency. This paper proposes a cylindrical cavity rubber layer local resonance double-layer plate phononic crystal unit structure based on relevant research on raised phononic crystals. The structural schematic is shown in Figure 1. The structure consists of two substrate plates, a scatterer, and two rubber layers. The rubber layer is treated with a cavity and is a cylindrical structure with a cylindrical cavity, while the scatterer is a solid cylinder. The upper and lower base plates are square plates.

The scattering body is a high-density metal, while the rubber layers are silicone rubber, and the substrate plate are epoxy resin, forming a “soft-hard-soft” resonance unit between the substrate plates. The structural and material parameters of the unit are presented in Table 1 and Table 2.

**Figure 2:**
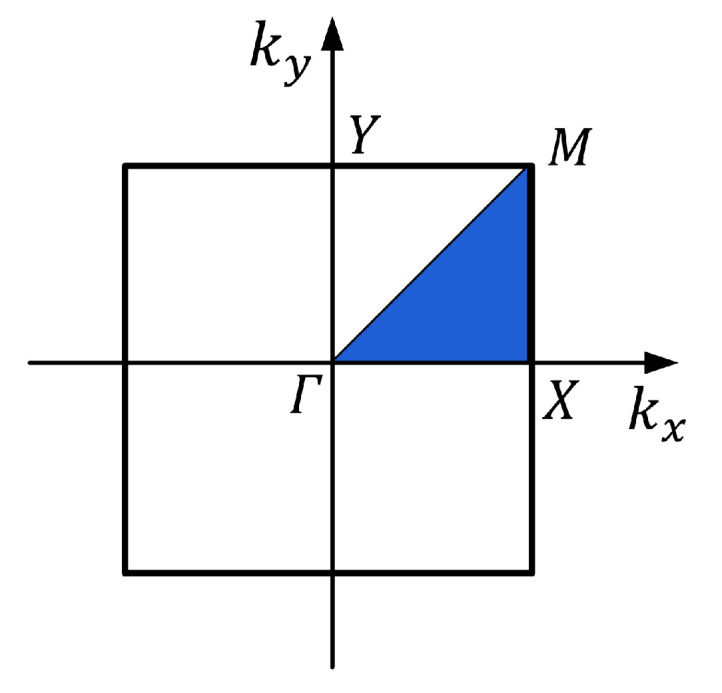
Diagram of irreducible Brillouin area.

The cylindrical cavity rubber layer phononic crystal structure shown in Figure 1 consists of a square symmetric lattice. The structure’s periodicity is analyzed only on the XOY plane since the phononic crystal unit’s periodic arrangement forms a phononic crystal plate. The corresponding irreducible Brillouin region is shown in the shaded part of Figure 2.

**Figure 3:**
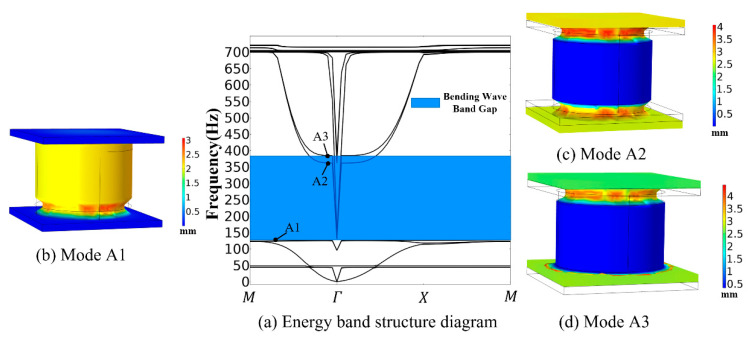
Energy band structure diagram and vibration mode (**a**–**d**).

The COMSOL finite element software can directly solve the band structure diagram of phononic crystals by setting periodic conditions. For the unit model in Figure 1, the corresponding boundary surfaces are selected and the Floquet periodicity condition is set. Then, the characteristic frequency is calculated and the energy band structure of the phononic crystal is acquired, as illustrated in Figure 3.

The bending wave band gap is generated by the vibration to the vertical direction. Notably, point A1 (127 Hz) corresponds to the band gap’s initiation frequency. The dominant vibration type of point A1(127 Hz) is vertical oscillation of the scatterer, while the upper and lower substrate plates remain static. Consequently, the vertical vibration generates a reaction force in the Z-direction, suppressing the transmission of bending vibration waves. The unit vibration modes of point A2 (362 Hz) and point A3 (384 Hz) are predominantly manifested as vertical vibration of the two substrate plates, while the scatterer oscillator remains stationary. The preceding reaction force on the substrate gradually decreases until it vanishes, resulting in the gradual transmission of the bending vibration wave. Notably, point A3 corresponds to the band gap’s cutoff frequency. From Figure 3, it is evident that the phononic crystal unit exhibits a bending wave band gap between 127 Hz and 384 Hz.

**Figure 4:**
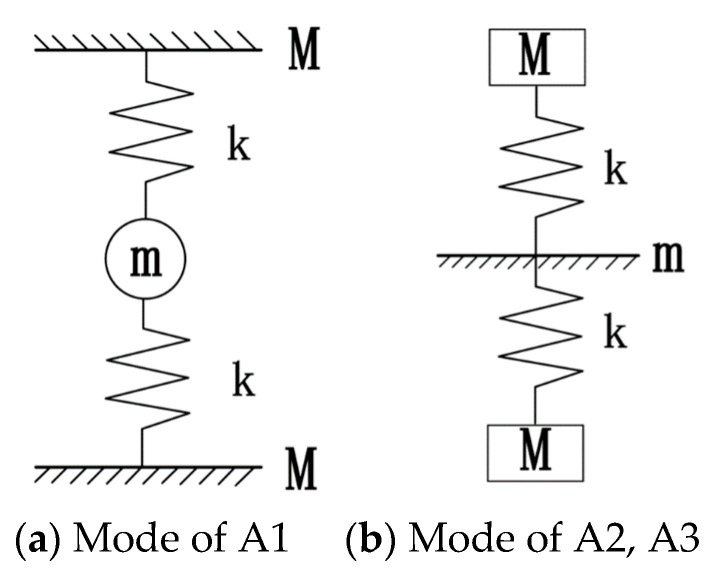
Unit simplified model (**a**,**b**).

In addition, a simplified model of the spring mass system is used to describe the three modes of vibration described above. For the mode of point A1, the matrix remains stationary and is simplified as mass M, the scattering body is simplified as mass m, and the rubber layer is simplified as stiffness k. The simplified model is shown in Figure 4a. At this point, the system characteristic frequency is $\sqrt{2k/m}$, which corresponds to the initial frequency and point A1. For the modes of points A2 and A3, the simplified model is shown in Figure 4b; the scatterer remains stationary; and the two substrates, rubber layers, and scatterers form two identical simplified models. At these two points, the system’s characteristic frequency is $\sqrt{k/M}$, which corresponds to the cutoff frequency. This indicates that the bending wave band gap is determined by the physical properties of substrate plates, scatterers, and rubber layers.

**Figure 5:**
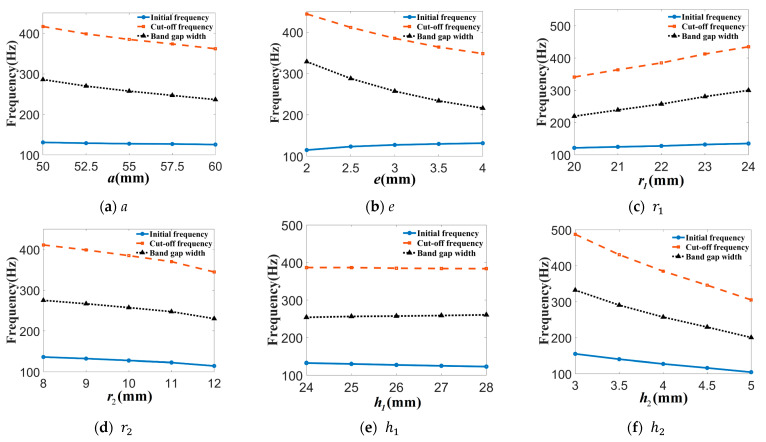
Influence of the parameters on the band gap of the bending wave (**a**–**f**).

Keeping the dimension of the phononic crystal structure as a constant, the control variable method was used to analyze the effect of each parameter on the bending wave band gap separately. Set the value of a∈{50,52.5,55,57.5,60}, e∈{2,2.5,3,3.5,4,4.5,5}, $r_{1}\in \{20,21,22,23,24,25\}$, $r_{2}\in \{8,9,10,11,12\}$, $h_{1}\in \{24,25,26,27,28\}$, $h_{2}\in \{3,3.5,4,4.5,5\}$, the unit is mm. The effect of each parameter on the band gap of the bending wave is shown in Figure 5.

From Figure 5, an increase in the radius cylindrical cavity $r_{2}$ and the height of the rubber layer $h_{2}$ will reduce the stiffness of the rubber layer, and an increase in scatterer height $h_{1}$ will reduce the mass of scatterer. Then, the initial frequency of the bending wave band gap will be reduced. A decrease in the lattice constants a and the substrate thickness e will reduce the mass of substrate plate. Then, the cutoff frequency of the bending wave gap will increase.

### 2.3. Vibration Transmission Characteristics of Phononic Crystal Plates

**Figure 6:**
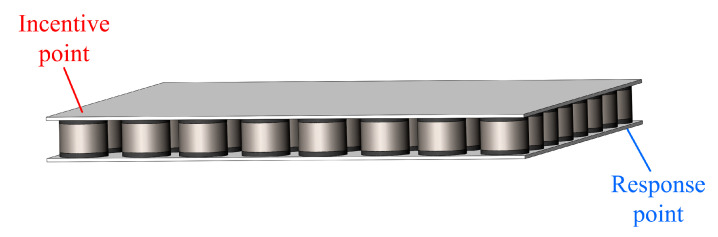
Diagram of phononic crystal plate.

Vibration transmission characteristics are important indicators for evaluating vibration reduction performance. Arranging the phononic crystal unit structure in Figure 1 with a finite number of periods, as depicted in Figure 6, the number of permutations in both the X and Y directions is 8, and the geometric size of the phononic crystal plate is 440 mm × 440 mm × 40 mm.

Figure 6 illustrates that one point (22.5 mm, 22.5 mm, 40 mm) is set as the excitation end, and another point (417.5 mm, 417.5 mm, 0) is set as the response end. The vertical point load excitation signal is applied, and the vertical acceleration signals at the excitation end and response end are extracted.

Vibration transmission loss is one of the important indicators for evaluating vibration reduction performance, defined as:

(7) $$TL=20{log}_{10}\left(\frac{\omega_{in}}{\omega_{out}}\right)$$

**Figure 7:**
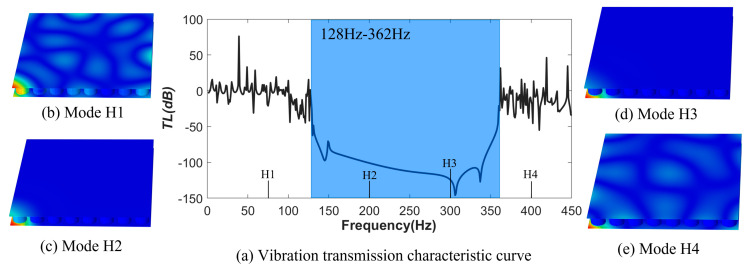
Bending wave vibration transmission curve of Phononic crystal plate (**a**–**e**).

In Equation (7), $\omega_{in}$ and $\omega_{out}$ represent the acceleration amplitude at the excitation and response points, respectively. Frequency domain simulation is performed on the system, and the bending wave vibration transmission curve of the crystal plate is depicted in Figure 7. It shows that the phononic crystal plate exhibits an excellent inhibitory effect on vibrations in the 128 Hz to 362 Hz range, which is in agreement with the calculated band gap range of 127 Hz to 384 Hz.

**Figure 8:**
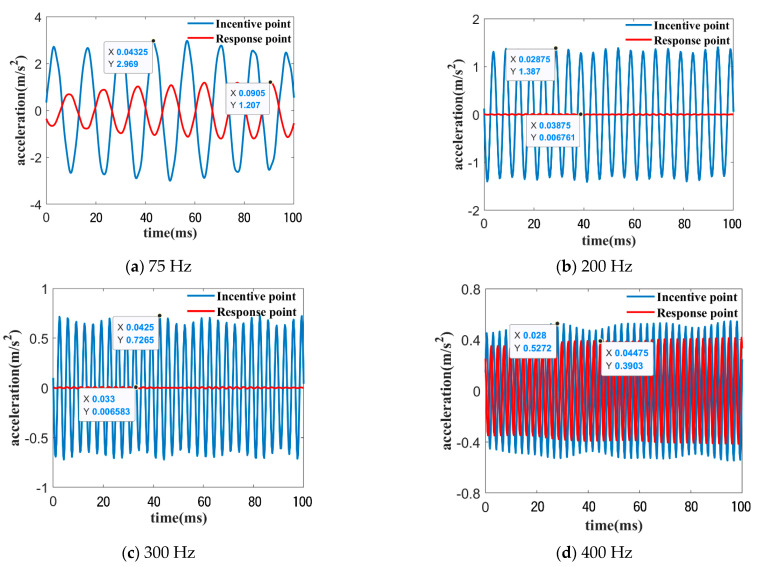
Acceleration curve under single frequency excitation at H1–H4 points (**a**–**d**).

Four characteristic frequency points, H1 (75 Hz), H2 (200 Hz), H3 (300 Hz), and H4 (400 Hz), are set up inside and outside the band gap. Figure 7b–e clearly indicates that bending vibration can be transmitted at the H1 and H4 frequency points outside the band gap, while bending vibration waves are well suppressed at the H2 and H3 frequency points inside the band gap. To further demonstrate the difference of bending vibration transmission at the inside and outside of the band gap, the excitation frequencies were set to four frequencies(H1–H4), and the phononic crystal plate was subjected to single-frequency excitation to obtain the curves of the acceleration values at the excitation and response points as a function of time, as shown in Figure 8. The corresponding input and output amplitude attenuation under H1~H4 excitation are −7.82 dB, −46.24dB, −40.86 dB, and −2.61 dB, respectively. The amplitude attenuation corresponding to the H2 and H3 frequencies located within the band gap is significantly greater than that of H1 and H4 outside the band gap.

## 3. Experimental Analysis of Vibration Transmission Characteristics

**Figure 9:**
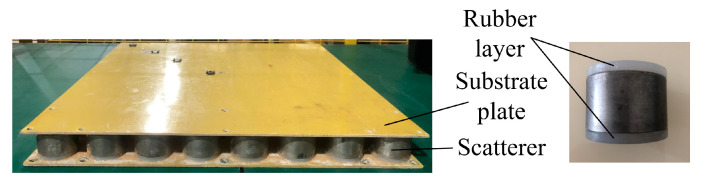
Experimental piece of phononic crystal plate.

To verify the correctness of the simulation analysis, the experimental piece was fabricated by machining the phononic crystal plate structure, as illustrated in Figure 9.

**Figure 10:**
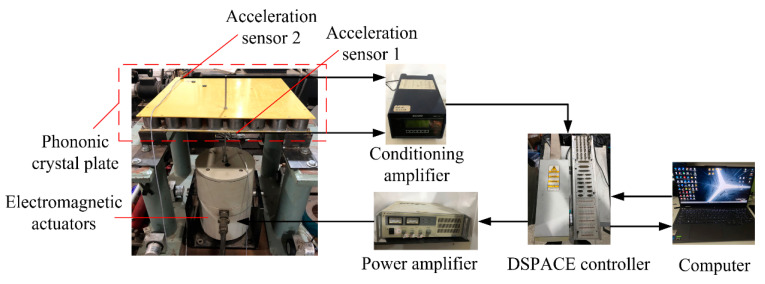
Experimental system for vibration transmission characteristics.

As illustrated in Figure 10, the experimental system of bending vibration characteristics was built according to the structural characteristics of phononic crystal plate. The plate’s four corners were fixed and connected to the base, and an electromagnetic exciter was installed. The experimental system was composed of a base, support seat, electromagnetic actuator, BK acceleration sensors, dSPACE controller, conditioning amplifier, power amplifier, and computer. The base was fixed to the foundation, and the support seat was connected to the base and the phononic crystal plate with screws to ensure proper connection between the plate and the base. An excitation signal was defined by the computer, compiled by the computer software, and fed into the dSPACE controller, and the vibration signal was exported by internal DA conversion. The vibration signal was passed through a power amplifier, which amplifies the signal and generates a signal that drives an electromagnetic actuator. The electromagnetic actuator generates a sinusoidal sweeping excitation force in the vertical direction on the phononic crystal plate through the top bar that is fixed to it, which is equivalent to a bending wave excitation on the crystal plate. The input signal of the excitation point (220 mm, 64 mm, 0) was obtained by using acceleration sensor 1, and the output signal of the response point (27.5 mm, 412.5 mm, 40 mm) was obtained by using acceleration sensor 2. These signals were amplified by the conditioning amplifier. The conditioning amplifier inputted the signal to the dSPACE controller, converted it into a digital signal via AD conversion and inputted it to the computer for storage, then converted the time domain signal into a frequency domain signal via fast Fourier transform.

**Figure 11:**
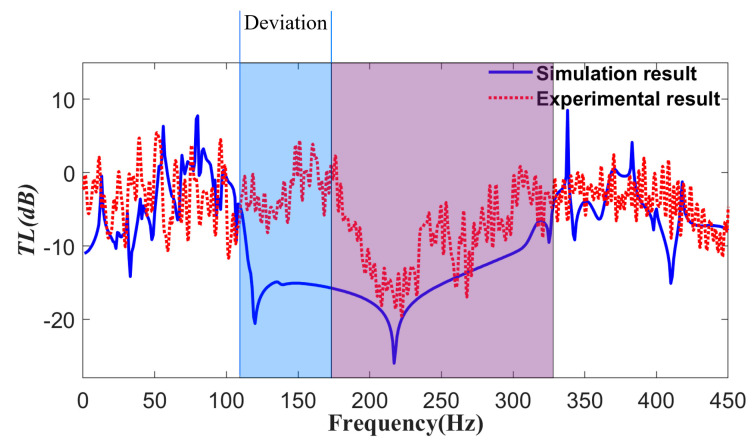
Experimental curve of vibration transmission characteristics.

The vibration transmission characteristic curves between the excitation and response points of the phononic crystal plate were obtained through experimental testing. In addition, an identical simulation model was constructed based on the boundary conditions of the experimental conditions, and the two results are compared in Figure 11.

Figure 11 shows that the phononic crystal plate exhibits significant suppression of bending vibration when the external excitation signal frequency ranges from approximately 175 Hz to 305 Hz, creating a curved wave band gap with a transmission loss of up to 20 dB around 220 Hz. The simulated bending vibration transmission curve indicates that the crystal plate approximately formed a bending wave gap in the range from 110 Hz to 315 Hz. The experimentally measured band gap range falls within the simulation range, but its bandwidth is slightly narrower than the simulated value.

**Figure 12:**
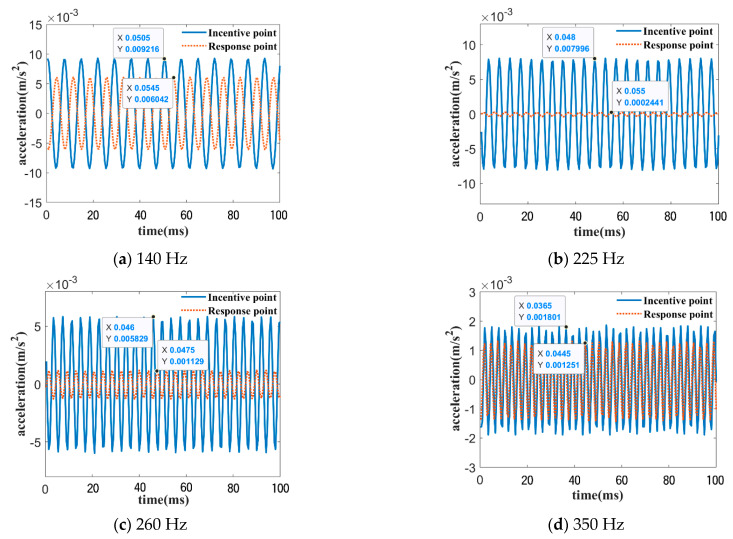
Input and output curves under single frequency excitation (**a**–**d**).

The characteristic frequencies were extracted and were 140 Hz, 225 Hz, 260 Hz, and 350 Hz inside and outside the band gap range of the bending wave experimental curve. Corresponding single-frequency excitation signals were applied, and the acceleration curves of the excitation and response points were obtained, as shown in Figure 12. The input and output amplitude attenuations for the four frequencies are 3.67 dB, 30.13 dB, 14.26 dB, and 3.16 dB, respectively. The amplitude attenuations for 225 Hz and 260 Hz within the band gap were significantly greater than those for 140 Hz and 350 Hz outside the band gap.

According to the experimental results shown in Figure 11 and the time domain curve in Figure 12, it is clear that the phononic crystal plate has a bending band gap with the vibration-damping features. However, the experimentally obtained bending wave band gap range of 175–305 Hz is slightly different from the simulated range of 110–315 Hz, which is within the simulation results. A modification was made to the simulation model, and the reasons for the discrepancy between the experiment and simulation were analyzed. Firstly, the manual bonding assembly caused the rubber layer scatterer composite oscillator to deviate from the substrate’s center, resulting in a higher initial band gap and a lower cutoff band gap. Secondly, the silicone rubber adhesive used for bonding has a higher bonding strength, resulting in a higher stiffness of the rubber layer, which was not considered in the simulation and which will cause a higher band gap range.

## 4. Parameter Optimization

### 4.1. RSM Analysis

The structural parameters of phononic crystals can impact their curved band gap range. Therefore, it is essential to further analyze how to efficiently and scientifically optimize the structural parameters to obtain a more suitable band gap range. Response surface methodology (RSM) analysis is an experimental design method that can fit the relationship and the fitted surface between each variable and the response variable. This method can effectively handle multivariate structure problems and guide structural design. Then, six structural parameters of phononic crystal element are taken as variables, keeping material parameters fixed, and the expressions between the initial and cutoff frequencies of the bending band gap and the structural parameters are fitted using RSM.

The values of a∈{50,55,60}, e∈{3,4,5}, $r_{1}\in \{20,22,24\}$, $r_{2}\in \{8,10,12\}$, $h_{1}\in \{24,26,28\}$, and $h_{2}\in \{3,4,5\}$ were set; the units are millimeters. The initial and cutoff frequencies are represented by F and N, respectively, and can be expressed as functions of six structural parameters, as shown in Equations (8) and (9), respectively. To investigate the impact of these parameters on the band gap range, we designed 54 experiments using three levels for each of the six factors. All experiments were simulated using finite element software, and the resulting data were analyzed by using RSM to create the expressions between the F, N and structural parameters.

(8) $$F\left(x\right)=\beta_{0}+\sum_{i=1}^{6}\left({\beta_{i}x}_{i}+{\beta_{ii}x}_{i}^{2}\right)+\sum_{i<j}\beta_{ij}x_{i}\cdot x_{j}$$

(9) $$N\left(x\right)=\alpha_{0}+\sum_{i=1}^{6}\left({\alpha_{i}x}_{i}+{\alpha_{ii}x}_{i}^{2}\right)+\sum_{i<j}\alpha_{ij}x_{i}\cdot x_{j}$$

Table 3 presents the fitting characteristic values of the response value F to analyze the degree of fitting. The R-squared value is 0.9983, and the surface’s predicted value closely matches the actual data, with a fitting degree of 99.83%. Furthermore, the Adeq precision value is 93.113, significantly greater than 4, suggesting that the analysis data are sufficient and can effectively guide the design process.

**Figure 13:**
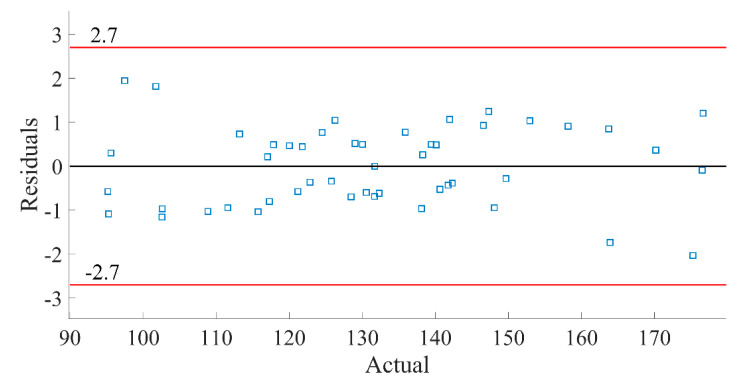
The residual curve between the true value and the predicted value of F.

**Figure 14:**
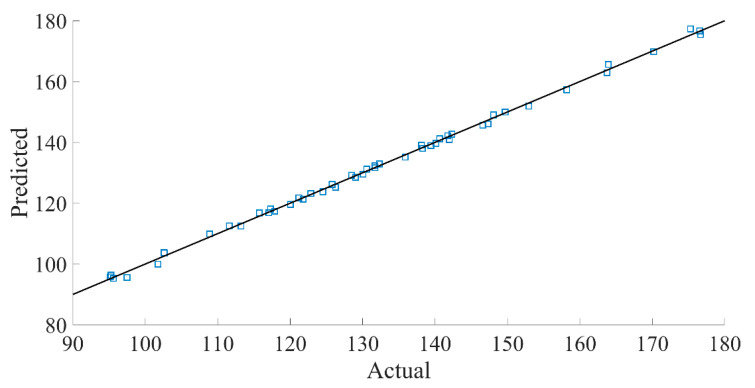
Real initial frequency and predicted value of F.

The precision and credibility of the regression model can be further illustrated by using residual analysis. Figure 13 displays the internal residual plot, indicating that all data points are distributed between (−3, 3), with 92.6% of the data between (−2, 2), which suggests a normal distribution. Figure 14 represents the relationship between the predicted and true values of the initial frequency F of the bent wave band gap, revealing a high degree of overlap between the predicted and true values, which further confirms the accuracy of the regression model.

The relationship between the response values *F, N*, and the six structural parameters obtained through RSM analysis is as follows:

(10) $$\begin{array}{ll} F= & 131.72-0.8237x_{1}+7.9308x_{2}-11.1941x_{3}+2.8052x_{4}-4.9466x_{5}-\\ & 26.6661x_{6}+1.0246{x_{1}}^{2}-0.7087{x_{2}}^{2}-1.4826{x_{3}}^{2}-1.3996{x_{4}}^{2}-\\ & 0.2187{x_{5}}^{2}+2.8689x_{6}^{2}-0.9763x_{1}x_{2}+0.894x_{1}x_{3}+1.0313x_{1}x_{4}+\\ & 0.1238x_{1}x_{5}+2.014x_{1}x_{6}+2.03x_{2}x_{3}+0.5637x_{2}x_{4}-0.2308x_{2}x_{5}-\\ & 0.919x_{2}x_{6}-1.0345x_{3}x_{4}+0.325x_{3}x_{5}+1.5776x_{3}x_{6}-0.1313x_{4}x_{5}-\\ & 1.652x_{4}x_{6}+1.089x_{5}x_{6} \end{array}$$

(11) $$\begin{array}{ll} N= & 348.05-22.3996x_{1}+43.9271x_{2}-29.3938x_{3}-32.9388x_{4}-\\ & 1.795x_{5}-79.3958x_{6}+4.3354{x_{1}}^{2}-1.1008{x_{2}}^{2}-3.4183{x_{3}}^{2}+\\ & 5.1979{x_{4}}^{2}-1.0796{x_{5}}^{2}+9.1529x_{6}^{2}-3.8375x_{1}x_{2}+2.6625x_{1}x_{3}+\\ & 2.4894x_{1}x_{4}-0.0513x_{1}x_{5}+10.365x_{1}x_{6}+2.6588x_{2}x_{3}-3.235x_{2}x_{4}+\\ & 0.0581x_{2}x_{5}-10.4625x_{2}x_{6}+1.8925x_{3}x_{4}+0.0362x_{3}x_{5}+\\ & 7.155x_{3}x_{6}-0.3263x_{4}x_{5}+8.3225x_{4}x_{6}+0.4575x_{5}x_{6} \end{array}$$

### 4.2. PSO Method

In this article, the goal is to achieve better vibration reduction and an effective working range for the phononic crystal structure. In order to meet low-frequency damping requirements, the initial frequency is usually maintained as low as possible while also maximizing the width of band gap. Therefore, the nonlinear programming equation is constructed with the band gap width as the optimization goal, as shown below:

(12) $${argmin-\left(F\left(x\right)-N\left(x\right)\right)}^{2}\;\begin{array}{c} s.t.\left\{\begin{array}{c} -1\leq x_{i}\leq 1,i=1,2,\ldots ,6\\ F\left(x\right)\leq F_{max}\\ N\left(x\right)\geq N_{min} \end{array}\right. \end{array}$$

**Figure 15:**
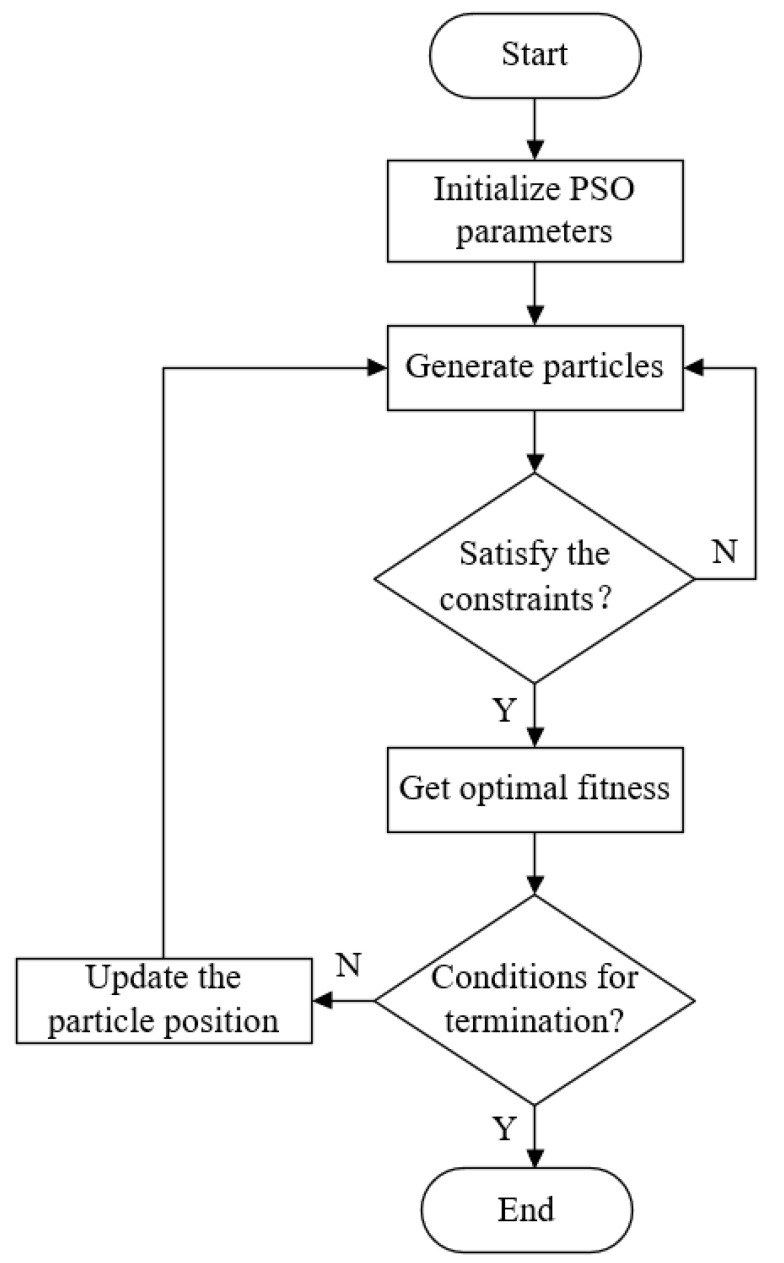
PSO-algorithm-solving structural parameters flowchart.

Equation (12) presents a typical nonlinear optimization problem. The simplex method or the interior point method can be used to obtain a solution that satisfies the objective function. However, due to the complexity of the optimization problem, these methods may only find a local optimal solution instead of the global one. A particle swarm optimization (PSO) algorithm, an alternative method, can be used to obtain the maximum band gap width by maximizing the structural parameters. This algorithm mimics the cooperative and informative behavior of birds and has become widely popular due to its ease of implementation and minimal number of parameters required [41]. Figure 15 gives a flowchart depicting the flow of the optimization search using the PSO algorithm. To achieve better vibration reduction and an effective working range, it is desirable for the band gap width to be as wide as possible while keeping the initial frequency low to meet low-frequency vibration reduction requirements.

**Figure 16:**
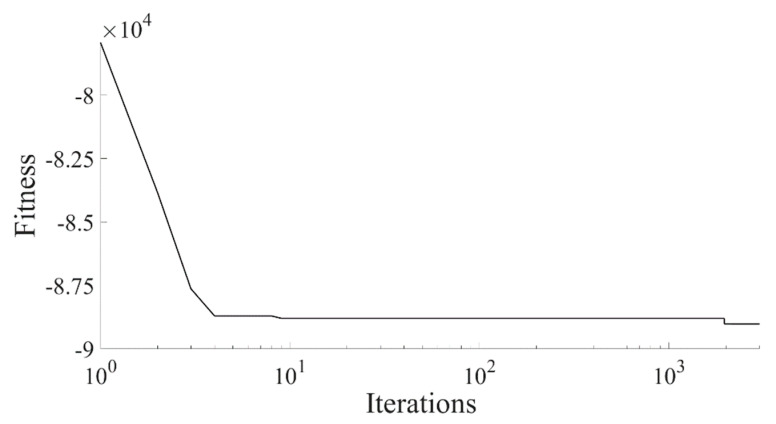
PSO algorithm iteration fitness.

The maximum band gap width and its corresponding structural parameters are separately solved by using the interior point method and a PSO algorithm. The PSO algorithm is configured with 500 particles, a maximum of 3000 iterations, and dynamic inertia weight. The two inertia weights are set to 1.5 and 0.7, respectively. The inertia weight decreases linearly with the number of iterations, and the learning factors are set to 1.5 and 2.0, respectively. In both methods, the cutoff frequency parameters in the constraint condition are set to $F_{max}$ = 120 Hz and $N_{min}$ = 380 Hz. The fitness curve obtained by the PSO algorithm is shown in Figure 16.

Figure 16 illustrates the fitness iteration curve, which indicates that the algorithm achieves convergence after approximately 2000 iterations, with a maximum fitness of approximately 89,016.41, corresponding to a band gap width of approximately 298.3562 Hz. The results obtained via the interior point method and the PSO algorithm for the maximum band gap width and structural parameters are presented in Table 4. To validate the model’s accuracy, we modeled the combination of parameters obtained using the interior point method and PSO algorithm; the simulation verification band gap values are presented in Table 5.

From Table 4, it is evident that the PSO algorithm produced a wider band gap parameter combination compared to the interior point method. The PSO algorithm achieved a maximum band gap width of 298.3562 Hz, while the interior point method yielded a maximum band gap width of 269.4683 Hz. Therefore, the PSO algorithm’s optimization accuracy is higher than the interior point method, and it is more suitable for phononic crystal structure parameter optimization. Moreover, Table 5 indicates that the band gap range optimized by the interior point method is 110.5317 Hz–380 Hz; the band gap range computed using the finite element model is 110.56 Hz–377.85 Hz. Similarly, the range optimized with the PSO algorithm is 120 Hz–418.3562 Hz. The range computed using the finite element model is 120.72 Hz–419.64 Hz. The optimized model’s band gap is very close to that obtained using the finite element model, further proving the regression model’s accuracy and credibility.

## 5. Conclusions

This article presents the design of a cylindrical cavity double-layer plate phononic crystal structure. Through finite element method analysis, the structure forms a bending wave band gap ranging from 127 Hz to 384 Hz. The mechanism of band gap formation is explained on the basis of vibrational modes. Furthermore, the article analyzes the vibration transmission characteristics and vibration damping performances of phononic crystal plates in both the frequency and time domains. An experimental system is constructed to verify the vibration damping performances of phononic crystal plates, and it analyzes the causes for deviation between simulation and experimental results. The article then applies RSM to obtain the response surface equations between the band gap and six structural parameters. In order to obtain the largest band gap width and lower initial frequency, the PSO algorithm is used to optimize these parameters, resulting in a band gap of 298.3562 Hz under the optimal parameter combination. The PSO algorithm outperforms the interior point method in obtaining a global optimal solution, wider band gap width, and corresponding structural parameters.

## Figures and Tables

**Table 1:** Structural parameters of unit.

Name	Parameter
Lattice constant a	55 mm
Scatter radius $r_{1}$	22 mm
Cylindrical cavity radius $r_{2}$	10 mm
Substrate thickness e	3 mm
Scatter height $h_{1}$	26 mm
Rubber layer height $h_{2}$	4 mm

**Table 2:** Material parameters of unit [40].

Component	Material	Density (kg/m^3^)	Elastic Modulus (Mpa)	Poisson’s Ratio
Substrate plate	epoxy resin	2000	4350	0.368
Rubber layer	silastic	1300	0.1175	0.469
Scatterer	steel	7850	210,000	0.3

**Table 3:** Characteristic values of the model.

Std. Dev	1.22	R-Squared	0.9983
Mean	131.76	Adj R-Squared	0.9965
C.V%	0.93	Pred R-Squared	0.9911
RRESS	203.14	Adeq Precision	93.113

**Table 4:** Optimization results obtained using two methods.

	Interior Point Method	PSO Method
a (mm)	50	50
$r_{1}\;(mm)$	24	24
$r_{2}\;(mm)$	12	12
e (mm)	3	3
$h_{1}\;(mm)$	28	28
$h_{2}\;(mm)$	4.5002	4.0984
F	110.5317 Hz	120 Hz
N	380 Hz	418.3562 Hz
Band gap width	269.4683 Hz	298.3562 Hz

**Table 5:** Optimization and verification results.

	Optimization Model F~N (Hz)	Simulation Verification F~N (Hz)
Interior point method	110.5317~380	110.56~377.85
PSO method	120~418.3562	120.72~419.64

## Data Availability

The data presented in this study are available on request from the corresponding author.
